# Metformin attenuate PTZ-induced apoptotic neurodegeneration in human cortical neuronal cells

**DOI:** 10.12669/pjms.333.11996

**Published:** 2017

**Authors:** Fehmida Bibi, Ikram Ullah, Myeong Ok Kim, Muhammad Imran Naseer

**Affiliations:** 1 Fehmida Bibi, King Fahd Medical Research Center (KFMRC), King Abdulaziz University, 21589, Jeddah, Saudi Arabia; 2 Ikram Ullah, Sulaiman Bin Abdullah Aba Al-Khail-Centre for Interdisciplinary Research in Basic Sciences (SA-CIRBS), International Islamic University, Islamabad, Pakistan; 3 Myeong Ok Kim, Department of Biology, College of Natural Sciences and Applied Life Science, Gyeongsang National University, Jinju, 660-701, Republic of Korea; 4 Muhammad Imran Naseer, Center of Excellence in Genomic Medicine Research, King Abdulaziz University, 21589, Jeddah, Saudi Arabia

**Keywords:** Neuroprotection, Metformin, HCN-2 Cortical cell line, Neuronal apoptosis

## Abstract

**Objective:**

Seizures are one of the neurodegenerative disorders of human being. Metformin has antioxidant properties and commonly used as an oral antidiabetic drug. The current study was aimed to observe the neuroprotective effect of metformin against PTZ-induced apoptotic neurodegeneration in human cortical neuronal cell culture.

**Methods:**

To observe that exposure of pentylenetetrazol (PTZ) at the dose of (30mM) for 30 minutes induced neuronal cell death by activation of caspase-3 in human cortical neuronal 2 (HCN-2) cell line. While the metformin at the dose of (20mM) along with PTZ for 30 minutes showed neuroprotection against PTZ-induced neuronal cell loss by MTT assay and Western blot analysis.

**Results:**

The results of this study showed that PTZ-induced neuronal cell death by activation of pro apoptotic proteins caspase-3 and 9 whereas the exposure of metformin showed its protective effect against neuronal loss in HCN-2 cell line. Finally, our results showed that exposure of metformin can prevent the harmful effect induced by PTZ in neuronal cells cultures.

**Conclusions:**

Our finding suggest that metformin exposure attenuates PTZ-induced neuronal cell death may act as a safe therapeutics and neuroprotective agent for the treatment of neuronal loss as result of seizure.

## INTRODUCTION

Epilepsy is among the utmost common neurological disorders affecting the structure of brain by inducing neuronal cell death.^1^ Pentylenetetrazol (PTZ) drug can cause seizure and results in cognitive disorders,^2^ changes in emotional behavior^3^ and neuronal loss.^4^ PTZ exposure cause brain damage and induce epileptic seizures by affecting specific receptors and the magnitudes of seizure differ in the developing brain as compared to the mature brain.^5^ A large number of signaling pathways are involved that results in seizure-induced neuronal cell damage, change in behavior, intellectual dysfunction and apoptosis.^6^

Metformin (N’, N’-dimethylbiguanide) hypoglycaemic drug used widely for the treatment of type 2 diabetes.^7^ Metformin not only involved in the reduction of blood glucose levels but also found to exert its beneficial effects in the cardiac diseases as well as stroke. Moreover it is reported recently in clinical trials that metformin meaningfully reduces the risk of stroke that are independent of its lowering the glucose effects.^8^ However, the fundamental molecular mechanisms how metformin effect remain largely unknown.

Neuron death occur in the seizure,^9^ However the mechanism of neuronal cell death after seizure remains unknown. Initiation of caspase-3 activation is known to be a crucial incident of apoptosis in the brain.^10^ It is well known that change in caspase-3 level in the brain changes the brain plasticity.^11^ It has been reported recently that metformin can interfere the apoptotic pathway by hindering the release of mitochondrial cytochrome-c and preventing neuronal loss and death.^12^ The clinical results are of great interest that by using metformin for both anti-hyperglycemic function and potent agent for the treatment of neurodegenerative disorders.

In this study we used a dose of PTZ (30mM for 30 minutes) to induce cell death in adult neuronal cell culture and metformin at the dose of 20mM to protect the effect of neuronal cells death. Our results suggested that exposure PTZ induce neurodegeneration, whereas, the treatment of metformin provide protection against the neuronal loos induced by PTZ can be used as therapeutic approaches for protection in adult neuronal cell culture.

## METHODS

### Cell culture and drug treatment

In this study we used HCN-2 cell line derived from the cortical tissue of the brain from the patients undergoing hemispherectomy for intractable seizures. This study was done during 2015 in King Abdulaziz University.

### Drug treatment

Primary HCN-2 cortical cells cultured for five days and further divided into groups for the drug treatments. These groups were divided as (1) Control contain DMEM medium (2) PTZ group containing PTZ (30mM) for 30min; (3) Metformin treated group with PTZ (30mM) and Metformin (20mM) for 30 minutes. Cells were used for the desired analyses at the end of the drug treatments.

### Measurement of Cell viability using MTT assay

HCN-2 cell growth after the drug treatment was measured by growth assays using 3-[4,5-dimethylthiazol-2-yl]-2,5-diphenyl tetrazolium bromide (MTT). HCN-2 cells were seeded into 96-well plates using 150 μl of media (control groups only DMEM media). Media with PTZ (30 mM) and Metformin (20 mM) for 30 minutes were incubated at 37°C. Cell viability was determined by using MTT (5 mg/ml in phosphate buffer saline, PBS) further incubated for 4 h at 37°C. Formazan dissolved in organic solvent was added in each well and plates were placed on shaker for 30 min in dark. Plates were read at 550 to 570 nm (L1) and 620 to 650 nm (L2) on scanning microplate reader spectrophotometer. The final optical density was used to calculate cell death and survival in each wells using formula as absorbance in drug treated wells divided by absorbance from control wells × 100%.

### Western blotting

Protein was extracted for the cultured neuronal cell after the drugs treatment. Each treatment group was repeated three times. The cells were homogenized in PBS (0.2 M) along with protease inhibitor. Bio-Rad protein assay was used for the measurement of protein concentration. SDS-PAGE gels was used for the separation of protein along with size markerand protein transferred to polyvinylidene difluoride (PVDF) membrane (Santa Cruz Biotechnology, Santa Cruz, CA, USA). Caspase-3 and nine primary antibodies were used. Detailed methodology was used as previously described.^13^

### Data analysis and statistics

Western blot data were analyzed by using Sigma Gel System (SPSS Inc., Chicago, IL). Density of values were expressed as mean ± SEM. Student‘s *t*-test was used for the comparisons between treated groups and control groups. Statistical significance was *P* < 0.05 in each case.

## RESULTS

### Effect of PTZ on cultured neuronal cell death

**Fig.1:**
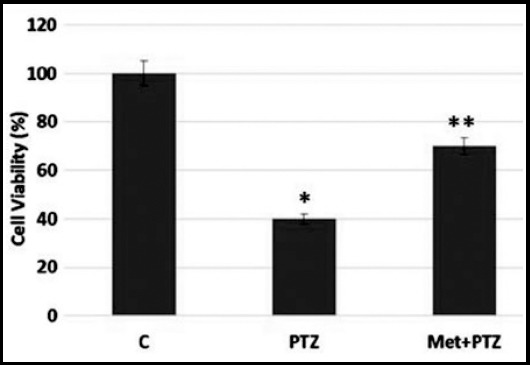
Cell viability was measured in HCN-2 cell cultures using MTT assay. After the exposure of drugs cell viability was measured in PTZ and Metformin treated groups. Data are the mean ± SE of three independent experiments (n = 3), with 3 plates in each experiment. Statistically significantly differences at P < 0.05 are indicated.

HCN-2 cortical neurons exposed with PTZ (30 mM) and Metformin (20 mM) in three groups for 30 minutes treated, and cell viability was measured by MTT assay. PTZ induced neuronal cell death and upon exposure of metformin reverse the effect of neuronal cell loss after 30 min as shown in Fig.1 as compared to the control group.

### Metformin protect against PTZ-induced apoptotic neurodegeneration

**Fig.2:**
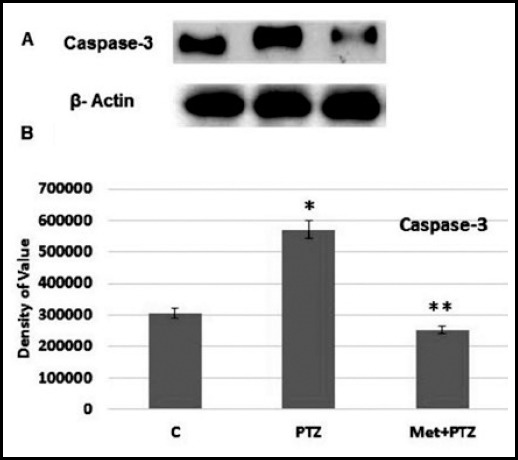
Western blot analysis was done after the drug treatment in HCN-2 adult neuronal cell culture. The caspase-3 antibody was used to identify the amount apoptotic protein in the culture. β-actin was as loading control. Density values, normalized to actin signals, are expressed as mean ± SD (n = 3) and are showed as arbitrary units. Significant different values are taken as P < 0.05.

**Fig.3:**
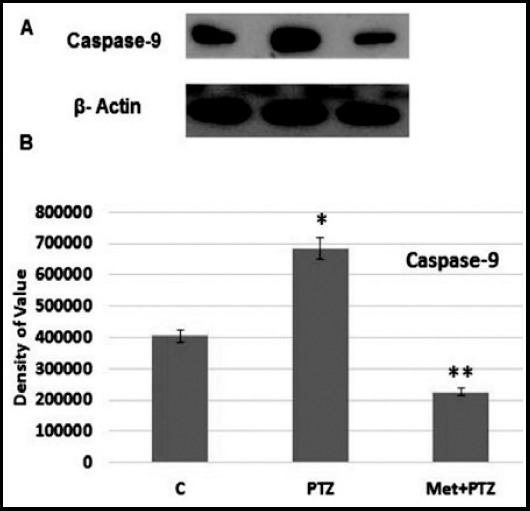
Western blot analysis was done after the drug treatment in HCN-2 adult neuronal cell culture. The caspase-9 antibody was used to identify the amount apoptotic protein in the culture. β-actin was as loading control. Density values, normalized to actin signals, are expressed as mean ± SD (n = 3) and are showed as arbitrary units. Significant different values are taken as P < 0.05.

Mitochondrial changes occurs after the activation of caspases pathway. In this study we observed that upon exposure of PTZ neuronal cell death starts significantly after activation of caspase-3 and 9. Caspases are proteases which play critical role in the initiation and execution of apoptotic cell death.^14^ The increased expression of caspase-3 is key player that activate the pathway leading to cell death including genomic DNA degradation.^15^ Further, the co treatment of metformin with PTZ can prevent PTZ-induced apoptotic neuronal loss by decreasing the expression of caspase-3 and 9. The doses of PTZ 30mM for 30 min induced neuronal cell death while metformin showed its protective effect by reversing the effect of PTZ as shown in the Fig. 2 and 3.

## DISCUSSION

In the present work, we have studied the neuroprotective effect of metformin against PTZ induced neurodegeneration. It was previously reported that PTZ induced neuronal cell death in prenatal rat hippocampal and cortical neurons.^16^ Metformin upon exposure with PTZ reverse the effect of neurodegeneration in HCN-2 adult cortical cells as we reported previously that vitamin C showed neuroprotection against ethanol induced cell death^17^ and PTZ-induced seizures in adult rats.^18^ It is also previously known that PTZ can induced epileptic seizures along with brain damage whereas the effect of seizure may be differ in the developing and in mature brain.^19^

Metformin is primarily used for patients with type 2 diabetes as first-line therapy as it can cross the blood brain barrier (BBB) rapidly^20^ and it has antioxidant property,^21^ along with anti-inflammatory,^21^,^22^ and neuroprotective^12^ effect as well. Moreover, in the animal model studies it showed positive effect against stroke also has beneficial effect for multiple sclerosis,^20^ protect ischemic brain and Alzheimer’s disease.^23^ Furthermore, Metformin was involved in the therapeutic benefits to prevent the formation and development of BBB breakdown^24^ as previously it was studied that PTZ exposure induced convulsive seizures along with increase of the BBB permeability in mice.^25^

In the present study, the increased expression of caspase-3 and 9 was studied upon exposure of PTZ in the HCN-2 neuronal cells. The protective effect of metformin against PTZ induced neuronal apoptosis was investigated using MTT assay and Western blot analysis. Our results showed that upon exposure of PTZ the neuronal loss occur by activation of caspase pathway and co treatment of metformin reverse the effect of PTZ induced apoptotic cell death in HCN-2 cell. Our results showed that administration metformin protect against PTZ-induced apoptotic cell death in HCN-2 cells. The mechanisms how metformin shows its neuroprotective effects further need to be elucidated, however, our results showed that treatment of metformin against PTZ are in agreement with neuroprotective actions of metformin as reported previously.^26^

In summary, our results presented that PTZ induced apoptotic neurodegeneration in HCN-2 neuronal cells. While the treatment of metformin decreased PTZ-induced apoptotic neurodegeneration suggested that metformin a drug used for diabetic a safe agent and may be used for the prevention and treatment of the apoptotic neurodegeneration and seizures.

## References

[1] Covolan L, Ribeiro LT, Long BM, Mello LE (2000). Cell damage and neurogenesis in the dentate granule cell layer in adult rats after pilocarpine- or kainite-induced status epilepticus. Hippocampus.

[2] Becker A, Grecksch G, Brosz M (1994). Naloxone ameliorates the learning deficit induced by pentylenetetrazol kindling in rats. Eur J Neurosci.

[3] Duncan JS (2002). Seizure-induced neuronal injury. Human data, Neurology.

[4] Pohle W, Becker A, Grecksch G, Juhre A, Willenberg A (1997). Piracetam prevents pentylenetetrazol kindling-induced neuronal loss and learning deficits. Seizure.

[5] Rauca C, Zerbe R, Jantze H (1999). Formation of free hydroxyl radicals after pentylenetetrazol-induced seizure and kindling. Brain Res.

[6] Engel T, Henshall DC (2009). Apoptosis, Bcl-2 family proteins and caspases: The ABCs of seizure-damage and epileptogenesis?. Int J Physiol Pathophysiol Pharmacol.

[7] Qaseem A, Humphrey LL, Sweet DE, Starkey M, Shekelle P (2012). Clinical Guidelines Committee of the American College of P. Oral pharmacologic treatment of type 2 diabetes mellitus: a clinical practice guideline from the American College of Physicians. Ann Intern Med.

[8] Cheng YY, Leu HB, Chen TJ, Chen CL, Kuo CH, Lee SD (2014). Metformin-inclusive therapy reduces the risk of stroke in patients with diabetes: a 4-year follow-up study. J Stroke Cerebrovasc Dis.

[9] McEachern JC, Shaw CA (1999). The plasticity-pathology continuum: defining a role for the LTP phenomenon. J Neurosci Res.

[10] Nicotera P (2000). Caspase requirement for neuronal apoptosis and neurodegeneration. IUBMB Life.

[11] Kolasa K, Harrell LE (2000). Apoptotic protein expression and activation of caspases is changed following cholinergic denervation and hippocampal sympathetic in growth in rat hippocampus. Neurosci.

[12] El-Mir MY, Dominique D, Gloria RV, Maria DE, Bruno G, Stephane A (2008). Neuroprotective role of antidiabetic drug metformin against apoptotic cell death in primary cortical neurons. J Mol Neurosci.

[13] Naseer MI, Shupeng L, Kim MO (2009). Maternal epileptic seizure induced by pentylenetetrazol: apoptotic neurodegeneration and decreased GABA B1 receptor expression in prenatal rat brain. Molecular Brain.

[14] Le DA, Wu Y, Huang Z, Matsushita K, Plesnila N, Augustinack JC (2002). Caspase activation and neuroprotection in caspase-3-deficient mice after in vivo cerebral ischemia and in vitro oxygen glucose deprivation. Proc Natl Acad Sci USA.

[15] Zhu C, Wang X, Hagberg H, Blomgren K (2000). Correlation between caspase-3 activation and three different markers of DNA damage in neonatal–cerebral hypoxia–ischemia. J Neurochem.

[16] Naseer MI, Ullah I, Rasool M, Ansari SA, Sheikh IA, Bibi F (2014). Down regulation of dopamine D1 receptors and increased neuronal apoptosis upon ethanol and PTZ exposure in prenatal rat cortical and hippocampal neurons. Neurological Sci.

[17] Naseer MI, Najeeb U, Ikram U, Zubair Hassan H, Yang MBC, Kim MO (2009). Vitamin-C protects ethanol induced apoptotic neurodegeneration in postnatal rat brain. Pak J Med Sci.

[18] Naseer MI, Ullah I, Ullah I, Lee HY, Cheon EW, Chung J (2011). Neuroprotective effect of vitamin C against PTZ induced apoptotic neurodegeneration in adult rat brain. Pak J Pharm Sci.

[19] Eracovic V, Zupan G, Varljen J, Simonic A (2003). Pentylenetetrazole induced seizures and kindling: changes in free fatty acids, superoxide dismutase, and glutathione peroxidase activity. Neurochem Int.

[20] Labuzek K, Suchy D, Gabryel B, Bielecka A, Liber S, Okopień B (2010). Quantification of metformin by the HPLC method in brain regions, cerebrospinal fluid and plasma of rats treated with lipopolysaccharide. Pharmacol Rep.

[21] Algire C, Moiseeva O, Deschênes-Simard X, Amrein L, Petruccelli L, Birman E (2012). Metformin reduces endogenous reactive oxygen species and associated DNA damage. Cancer Prev. Res.

[22] Hattori Y, Suzuki K, Hattori S, Kasai K (2006). Metformin inhibits cytokine-induced nuclear factor jB activation via AMP-activated protein kinase activation in vascular endothelial cells. Hypertension.

[23] Li J, Benashski SE, Venna VR, McCullough LD (2010). Effect of metformin in experimental stroke. Stroke.

[24] Takata F, Dohgu S, Matsumoto J, Machida T, Kaneshima S, Matsuo M (2013). Metformin induces up-regulation of blood–brain barrier functions by activating AMP-activated protein kinase in rat brain microvascular endothelial cells. Biochem Biophys Res Commun.

[25] Danjo S, Ishihara Y, Watanabe M, Nakamura Y, Itoh K (2013). Pentylenetrazole-induced loss of blood-brain barrier integrity involves excess nitric oxide generation by neuronal nitric oxide synthase. Brain Res.

[26] Ullah I, Ullah N, Naseer MI, Lee HY, Kim MO (2012). Neuroprotection with metformin and thymoquinone against ethanol-induced apoptotic neurodegeneration in prenatal rat cortical neurons. BMC Neuroscience.

